# Bleaching Teeth

**Published:** 1881-06

**Authors:** James Truman

**Affiliations:** Philadelphia, Pa.


### ARTICLE VII.

### *Bleaching Teeth.*

BY JAMES TRUMAN, D. D. S., PHILADELPHIA, PA.

The repeated inquiries in regard to the best modes to be adopted to change the color of teeth having devitalized pulps is an inducement to revive the process that I adopted some twenty years ago, and gave to the profession in the October number of the *Dental Times*, Vol. II., 1864, and subsequently in a second article, published in the same journal, Vol. VI., July, 1868. As so many years have elapsed since that period, and as a new generation of workers have come on the stage, it seems not only appropriate but necessary that the ideas then inculcated should be republished for their instruction. An additional incentive to this course is found in the fact that a very meagre statement of this process has recently been published in the foreign journals as a new mode of treatment; and, as this is very imperfectly given, practice under it must not only bring the process into disfavor, but is very likely to lead to disastrous results.

The bleaching of teeth is by no means a new idea. It was the subject of frequent experiments by some of the earlier operators, but with only partial success. The interest that environed it, and the necessity that we should have a better mode to remove these unsightly organs, led me into a series of long-continued experiments, with the hope of evolving a process worthy of adoption. It was soon found that any operation, to be of practical value, must go beyond anything previously accepted, and must, also, run in opposition to long-established prejudices. This led to delay in publishing the conclusions, and it was not until several years had elapsed, and doubt no longer existed as to its value, that it was given as the simplest, and, in the judgment of the writer, the only effectual means of accomplishing this object. Seventeen years have elapsed, and the opinion then expressed has not been altered, nor has there been anything better offered to accomplish this desired result. That but little interest was manifested was natural. It was the adoption of radical means to overcome a great difficulty, and the average human mind does not take kindly to anything or any process that upsets established modes of thought or practice. It has, however, outlived that period, and has become, in the practice of many, an operation regularly performed whenever required.

It is unnecessary to enter into the character of discoloration in detail; it is well understood, and may be briefly classified under three heads.

1st. That produced by the destruction of the pulp vitality from any of the well-known causes, as from blows, attacks of caries, etc., the subsequent transfusion of the decomposed tissue through a portion of the tubuli lying near the surface of the cavity of decay giving the tooth a dingy-blue appearance. This may occur in teeth affected by caries or in those without external evidence of disease.

2d. The more aggravated cases, from the same causes, where the whole tooth has become so much discolored as to require active treatment for the removal of the disfigurement.

3d. Of the latter we have teeth of a dingy-yellow, with gums in an inflamed condition; root much roughened; tooth somewhat loose in the alveolus; periosteum partially destroyed, and the lesion accompanied at times by a discharge of pus.

In the treatment, these three conditions must be carefully considered, as the prognosis will depend largely upon them. It is evident that the first is merely a superficial discoloration, the most of which can be generally removed with the excavator or drill. The subsequent filling with gold, oxy-chlorides, or zinc-phosphates will change the color sufficiently without resorting to the bleaching process. Indeed, the latter is only really required for the second class of teeth, for those of the third have become so thoroughly diseased that attempts to restore them can only be regarded as a partial success; but, as no tooth should be lost without an effort, these are not necessarily thrown out of the list of salvable teeth.

*Treatment.*—The fact that teeth with devitalized pulps, however produced, are always liable to be involved in acute inflammatory conditions, must be recognized as an important factor in any treatment attempted. The utmost care must therefore be exercised in the preliminary operations, to avoid unnecessary irritation. The removal of all remains of decomposed pulp from the canal is of vital importance; but this must not be performed in a rapid, careless manner, as a tooth so circumstanced is extremely sensitive to all external impressions. It is of great moment that no inflammation of the periosteum should supervene, as that, necessarily, not only complicates the operation, but renders it more doubtful of success. The rule to be remembered is, that in proportion to the vitality of the tooth will be the ratio of good results in bleaching.

The removal of the pulp should be followed by a thorough washing of the canal with a five per-cent. solution of carbolic acid. Anything stronger than this may become in itself an irritant,—a fact seemingly forgotten by many who make use of this remedy on all occasions in its full strength. There may be times when this will be required, but they are exceptional, and it certainly is not indicated here.

If this preliminary process has been satisfactorily conducted, the next operation may be proceeded with, viz., that of filling the canal as its *upper third.* This should be done with gold, and must be most thoroughly performed. The agent subsequently brought into use is extremely irritating and very penetrating, hence all avenues of egress to the periodonteum must be effectually closed. The question may be asked, why fill only the upper third? Because it is absolutely necessary for success that the root shall be bleached as well as the crown. · A failure to remember this will possibly result in a re-discoloration, from causes to be named hereafter. It must be remembered that the pulp-chamber in the crown requires the same careful treatment as that given to the canal. It must be thoroughly cleaned of all *debris* to its fullest extent, and that in the incisor and canine teeth is almost to the enamel line of the cutting-edges. Failure in this operation here will necessarily result disastrously. I prefer, after cleaning the canal, to follow it with the drill, in order to cut away a portion of the dentine, and also to remove, with the excavator or drill, a portion of the same tissue in the crown. Having proceeded thus far, the case is prepared for the further process of bleaching.

[TO BE CONTINUED.]

